# Effect of a Milk-Based Fruit Beverage Enriched with Plant Sterols and/or Galactooligosaccharides in a Murine Chronic Colitis Model

**DOI:** 10.3390/foods8040114

**Published:** 2019-04-04

**Authors:** Gabriel López-García, Antonio Cilla, Reyes Barberá, Amparo Alegría, María C. Recio

**Affiliations:** 1 Nutrition and Food Science Area, Faculty of Pharmacy, University of Valencia, Avda. Vicente Andrés Estellés/n, 46100 Burjassot (Valencia), Spain; gabriel.lopez@uv.es (G.L.-G.); reyes.barbera@uv.es (R.B.); amparo.alegria@uv.es (A.A.); 2 Department of Pharmacology, Faculty of Pharmacy, University of Valencia, Avda. Vicente Andrés Estellés/n, 46100 Burjassot (Valencia), Spain; maria.c.recio@uv.es

**Keywords:** milk-based fruit beverage, plant sterols, galactooligosaccharides, mice, chronic ulcerative colitis

## Abstract

The potential anti-inflammatory effect of plant sterols (PS) enriched milk-based fruit beverages (PS, 1 g/100 mL) (MfB) with/without galactooligosaccharides (GOS, 2 g/100 mL) (MfB-G) in an experimental mice model of chronic ulcerative colitis was evaluated. Beverages were orally administered to mice every day by gavage to achieve PS and GOS doses of 35 and 90 mg/kg, respectively, and experimental colitis was induced by giving mice drinking water *ad libitum* containing 2% (*w*/*v*) dextran sulphate sodium (DSS) for 7 days, alternating with periods without DSS up to the end of the study (56 days). MfB beverage showed significant reduction of symptoms associated to ulcerative colitis and improved the colon shortening and mucosal colonic damage, but it was not able to reduce the increase of myeloperoxidase levels produced by DSS. MfB-G showed higher incidence of bloody feces and loss of stool consistency than MfB, as well as high levels of immune cells infiltration in colon tissue and myeloperoxidase. Therefore, PS-enriched milk-based fruit beverage could be an interesting healthy food to extend the remission periods of the diseases and the need to evaluate, in a pre-clinical model, the anti-inflammatory effect of the combination of bioactive compounds in the context of a whole food matrix.

## 1. Introduction

Ulcerative colitis (UC) is a chronic inflammatory disease, within intestinal bowel diseases (IBD), characterized by a tissue destruction of large intestine with relapsing phases followed by periods of remission [1]. The most common treatment strategies used include pharmacological therapy (steroidal and non-steroidal drugs) or, in the worst cases, removing portions of affected gastrointestinal tract through surgery. Although, pharmacological therapy has shown efficacies in ameliorating the severity and symptoms of IBD, typically does not lead to long periods of remission. Consequently, recent investigations have focused on the study of the impact of diet and healthy foods as potential alternative, and even the possible combination of nutraceuticals and healthy foods with pharmacological therapy in cases when the patient is not eligible for conventional therapy which could help extend remission periods and improve life quality of IBD patients [2,3,4,5,6].

A recent meta-analysis has associated a high intake of fruit and vegetables with a reduction of UC in European population, suggesting that the intake of their bioactive compounds (fiber, antioxidant vitamins and phytochemicals such as polyphenols, carotenoids, isoflavones) could explain this inverse association [7]. In murine colitis models, which mimic human pathology, it has been demonstrated the preventive effect on pro-inflammatory intestinal process associated to the intake of mango [8] or pineapple [9] juices, polyphenols-rich extracts from orange [10] or its sub-products [11], apple [12] and pomegranate [13]. Polyphenols seems to be the main bioactive compounds involved in the anti-colitic action of fruits, due to its ability to inhibit some pivotal pro-inflammatory mediators as nuclear transcriptional Factor-κB (NF-κB) and specific cytokines (Tumor Necrosis Factor-α (TNF-α), and interleukin 1-β (IL-1β)) and the induction of antioxidant defence systems. Furthermore, lipophilic compounds present in vegetables and fruits, such as fat-soluble vitamins (pro-vitamin A) [14], carotenoids (β-carotene) [15] and plant sterols (PS) [16,17,18,19,20,21], have displayed important anti-inflammatory and inmmunodulatory effects. In particular, PS could be a good strategy to prevent IBD, because they show a very little absorption in the intestine (0.5–2%) and reach the colon, where could exert anti-inflammatory effects. Moreover, the European Union authorized PS addition into milk-based fruit beverages [22], among other foods, to achieve the necessary amount of 1.5–3 g PS/day (not attainable with normal diet) for the well-known cholesterol-lowering effect [23]. PS have shown promising results in animal colitis models induced by trinitrobenzene sulfonic acid (TNBS) [16,17], dextran sodium sulphate (DSS) [18,19,20,21,22,23,24] or high-fat diet (40–60%) [19,20,21]. Doses between 20–150 mg/Kg/day help to mitigate some important parameters associated to UC such as symptoms, colon shortening and presence of edema, histopathology and myeloperoxidase (MPO) activity on colon tissue.

Other bioactive compounds that have gained attention are prebiotics, which stimulate selectively beneficial intestinal bacteria, helping to maintain intestinal mucosal barrier and enhance defense against pathogenic microorganism [25]. In this sense, galactooligosacharides (GOS) have been proposed as an active ingredient to prevent or alleviate symptoms associated with IBD [26]. Human clinical trials have demonstrated that consumption of GOS contained in chocolate or banana flavored chews (3.5–7 g/day) [27] and sachets (5.5 g/day) [28,29], reduce different pro-inflammatory markers such as calprotectin in feces, plasma C-reactive protein [28] and cytokines (IL-6, IL-1β and TNF-α) [29], with a concomitant increase in levels of fecal secretory immunoglobulin A (sIgA) [28] and reduction of common ulcerative colitis symptoms [27]. However, in animal studies the effect of GOS on colitis is controversial. GOS (4 g/Kg/day) administration for 10 days increase bifidobacterial number in colon in a TNBS colitis induced murine model, but it was not linked with a reduction of pro-inflammatory markers as MPO activity and colon damage [30]. On the other hand, in mice smad-3 feed with GOS (5 g/Kg/day) for 42 days an improvement of colitis severity and increase of natural killer activity were observed after colitis induction by *Helicobacter hepaticus* [31].

Enrichment of healthy foods with bioactive compounds could be an effective strategy to help mitigate the symptoms associated with IBD and extend remission periods [3]. In this sense, milk-based fruit beverages could be a suitable nutritional matrix for this purpose due to its low-fat content and good sources of bioactive compounds such as polyphenols, carotenoids and vitamins. Moreover, previous studies of our research group have demonstrated the beneficial effect of PS enriched milk-based fruit beverages on oxidative stress prevention and intestinal epithelia integrity maintenance in Caco-2 cells [32], as well as, their systemic anti-inflammatory properties, in hypercholesterolemic post-menopausal women, through the serum increase of anti-inflammatory cytokine IL-10 with concomitant reduction of IL-1β, cytokine which play an important role on the development of IBD [33]. 

The present study investigates for the first time, the effect of the daily intake of a healthy food, as milk-based fruit beverages, enriched with bioactive compounds (PS and/or GOS) on clinical symptoms and inflammatory process of UC, using a pre-clinical chronic murine model induced by DSS. The aim of this preliminary study is to evaluate if PS enriched milk-based fruit beverages with/without GOS are a suitable dietetic strategy to help to mitigate the health problems associated with UC. 

## 2. Materials and Methods 

### 2.1. PS Milk-Based Fruit Beverage Formulation

Two Ps-enriched (1 g/100 mL beverage) milk-based fruit beverages were used: MfB and MfB-G, without or with addition of GOS (1.8 g/100 mL beverage), respectively. Both beverages contained skimmed milk with the addition of milk fat and whey protein concentrate enriched with milk fat globule membrane (MFGM, Lacprodan^®^ MFGM-10 from Arla Foods Ingredients, Sønderhøj, Denmark), mandarin juice from concentrate (45%) (Source of β-cryptoxanthin 205 µg/100 mL beverage) and banana puree (4%). PS were added during beverage formulation as microencapsulated free microcrystalline PS (β-sitosterol 80%; sitostanol 12%; campesterol; 7%; campestanol 1% and stigmasterol 0.7%) from tall oil in a powder form (Lipohytol^®^ 146 ME Dispersible from Lipofoods, Barcelona, Spain). GOS syrup (Vivinal^®^ GOS from Friesland Campina Ingredients, Amersfoort, Netherland) containing approximately 59% GOS, 21% lactose, 19% glucose and 1% galactose based on dry matter. In order to guarantee microbiological stability, beverages were subjected to pasteurization (90 °C for 30 s) and filled aseptically in 250-mL tetra bricks. 

### 2.2. Animals and Treatment

Female C57BL/6 mice, aged 6–8 weeks with weights ranging between 18 to 20 g, provided by Harlan Interfauna Iberica (Barcelona, Spain) were used in this study. Animals were acclimatized for 7 days under a 12-h light/ dark cycle at 22 °C/60% humidity and fed with a standard laboratory rodent diet and water *ad libitum* before experiments. All experiments were approved by the Institutional Ethics Committee of the University of Valencia, Spain (2017/VSC/PEA/00143).

**Figure 1:**
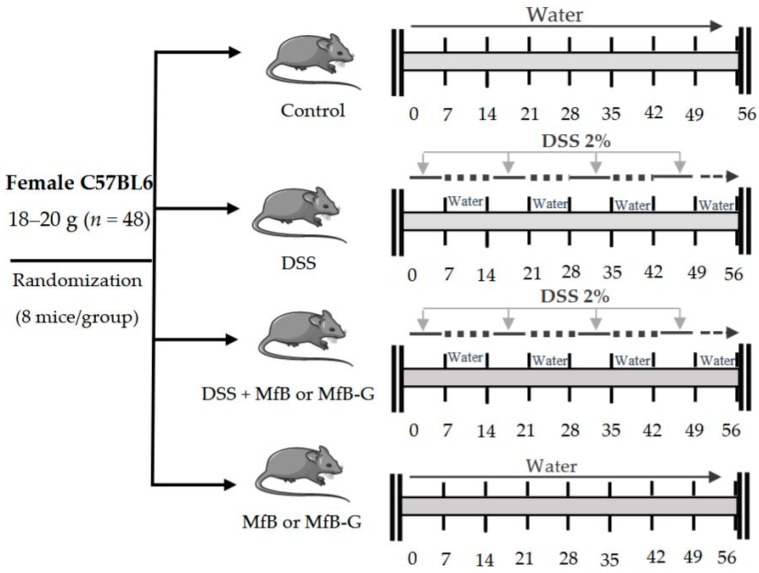
Experimental design of the chronic colitis induction by dextran sulphate sodium (DSS) (2%, *w*/*v*) and plant sterol enriched milk-based fruit beverages without (MfB) or with galactooligosacharides (MfB-G) administration in the procedures.

Chronic colitis induction was performed by repeated administration of a 2% (*w*/*v*) of DSS (in drinking water) for 7-day cycles according to Marín et al. [34] The DSS phases were interrupted by 7-days cycles of access to drinking water without DSS. MfB and MfB-G were orally administered everyday by gavage (0.15 mL) to achieve 35 and 95 mg/Kg body weight for PS and GOS, respectively (see Figure 1). Animals were randomly assigned into six intervention groups with 8 mice/group (*n* = 48): control (mice received water during all experiment), DSS (access to drinking water containing 2% DSS), DSS + MfB (drinking water containing 2% DSS + 0.15 mL of MfB), DSS + MfB-G (drinking water containing 2% DSS + 0.15 mL of MfB-G), MfB (received only 0.15 mL of MfB) and MfB-G (received only 0.15 mL of MfB-G). 

Water intake was monitored three times a week to guarantee equitative DSS intake among different colitis mice groups and the type of beverages did not affect drink behavior. No statistically significant differences (*p* > 0.05) in water intake was found among different mice group (ml/mouse/day); control (4.3 ± 0.5), DSS (4.2 ± 0.8), DSS + MfB (4.5 ± 0.7), DSS+MfB-G (4.8 ± 1.1), MfB (4.6 ± 0.4) and MfB-G (4.5 ± 0.5).

### 2.3. Disease Activity Index (DAI)

The disease activity index (DAI) was determined according to the parameters outlined in Table 1. Animal body weights, stool consistency and visible blood in feces were recorded three times a week during all experiments. Stool consistency and blood in feces parameters were evaluated checking fresh feces contained in each animal group cage and scored following the method described by Marín et al. [34]. Also, blood presence around mouse perianal area was taken as indicator of blood in feces. DAI was calculated by combining scores (WS: weight loss; SC: stool consistency; BF: bloody feces) following the next formula DAI: (WS + SC + BF)/3 according to the methodology described by Marín et al. [34].

### 2.4. Colon Shortening and Presence of Edema

Animals were sacrificed at day 56 by cervical dislocation and their colons (ileo-cecal junction to anal verge) were removed. Fecal residues were washed with cold phosphate buffered saline (PBS) and the length and weight were measured. Presence of edema in colonic tissue was evaluated working out the weight/length (mg/cm) ratio. Then, colons were cut in four portions of approximately 2 cm and frozen immediately at −80 °C until use. 

### 2.5. Histological Analysis

Portions of approximately 2 cm from the colon of every group of mice were fixed in freshly prepared 4% formaldehyde in PBS buffer (pH 7.2), embedded in paraffin, and sectioned at 4 µm. Sections were stained with hematoxylin and eosin for histopathological analysis and examined by light microscopy [35]. The slides were analysed and scored as previously described Cooper et al. [36] according to the criteria listed in Table 2. Scores were calculated by adding the score for the three parameters, giving a maximum score of 10.

### 2.6. Myeloperoxidase (MPO) Assay

In the model of experimental colitis, neutrophil infiltration into the colon was assessed indirectly by measuring myeloperoxidase (MPO) following the method elaborated by Bradley et al. [37]. Colon samples were weighed and blended in homogenizer with phosphate buffer (0.22 M) containing 0.5% of hexadecyl-trimethyl-ammonium bromide. Samples were centrifuged at 12000 g/4 °C/20 min and supernatants were placed on 96-well plates. Estimation of MPO content in colonic tissues was evaluated spectrophotometrically (450 nm) by measuring the 3,3′,5,5′-tetramethyl-benzidine (MPO substrate) oxidation in presence of H_2_O_2_ [34].

### 2.7. Statistical Analysis

The results are expressed as means ± standard deviation values (*n* = 8 for each group). One-way analysis of variance (ANOVA) followed by the Tukey post-hoc test was applied to determine differences among treatments. A significance level of *p* < 0.05 was adopted for all comparisons, and the Statgraphics Centurion XVI.I statistical package (Statpoint Technologies Inc., VA, USA) was used throughout.

## 3. Results

### 3.1. Evaluation of Clinical Symptoms of Induced Chronic Colitis in Mice

**Figure 2:**
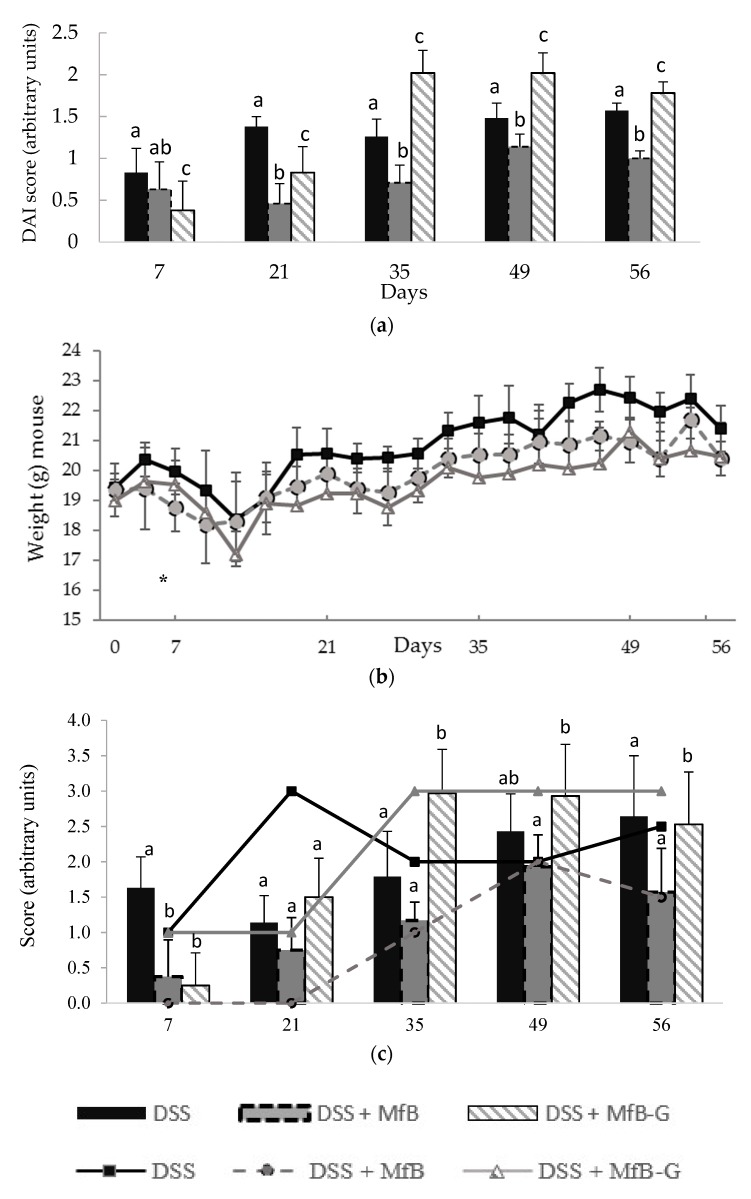
Effect of plant sterol enriched milk-based fruit beverages without (MfB) or with galactooligosacharides (MfB-G) on clinical symptoms in DSS-induced chronic colitis model. (**a**) Disease activity index (DAI) score after DSS (dextran sulphate sodium) administration in each cycle; (**b**) body weight per mouse during all the experiments; (**c**) stool consistency loss (lines) and presence of blood in feces (bars) at the end of each DSS administration cycle. Bars/markers show the mean ± standard deviation (*n* = 8). Different lowercase letters (a–c) indicate statistically significant differences (*p* < 0.05) among mice groups (DSS, DSS + MfB and DSS + MfB-G) within the same DSS cycle. (*) Denotes statistically significant differences (*p* < 0.05), compared to the DSS group, at the same day.

Mice exposed to DSS in drinking water (2% *w*/*v*) showed marked clinical symptoms (DAI values) during all experiment, being slightly lower in the first DSS cycle (7 days) (see Figure 2a). Administration of MfB significantly reduced DAI values between 24–67% compared to DSS group, with the exception of the first DSS cycle. MfB-G beverage attenuated DSS derived symptoms during the first cycles (7 and 21 days), reducing DAI values between 40–54% with respect to the DSS group. However, from the third DSS cycle (35 days) onwards, an increase of DAI (1.6 fold compared to the DSS group) was observed. With the aim to evaluate the effect of beverages on specific illness derived symptoms, Figure 2b,c show the evolution of mice weight, stool consistency and presence of blood in feces, respectively. In general, MfB and MfB-G administration did not have any differences in terms of weight loss compared to DSS group during the study, with the exception of MfB-G beverage, where a statistical (*p* < 0.05) higher weight loss at 10 day (20% vs. DSS group) was observed. Both beverages statistically (*p* < 0.05) reduced the presence of blood in feces (4 to 6-fold) during the acute phase of the illness (7 days) with respect to the DSS group (Figure 2c). However, in subsequent DSS cycles, this protective effect was not observed with both beverages and sometimes the incidence of blood in faces increased in the MfB-G mice group (third and fifth cycle). With respect to stool consistency, MfB prevented completely the appearance of diarrhea during the first two DSS cycles (7 and 21 days) and reduced up to 50% their incidence during the third DSS cycle, compared to DSS group, (see Figure 2c). However, after fourth (49 days) DSS cycle MfB did not show differences in the stool consistency in comparison to DSS group, although a slight beneficial effect was observed at the end of the study. On the other hand, MfB-G partially prevented the loss of stool consistency during the second DSS cycle, but this loss rapidly increased in the subsequent DSS cycles after which stool consistency remained constant for the rest of the study. The loss of stool consistency observed in the MfB-G group during the third cycle coincides with the increase of blood presence in feces. This suggests that the higher acute diarrhea observed is related to colonic epithelium erosion. 

### 3.2. Colon Length Shortening and Presence of Edema

**Figure 3:**
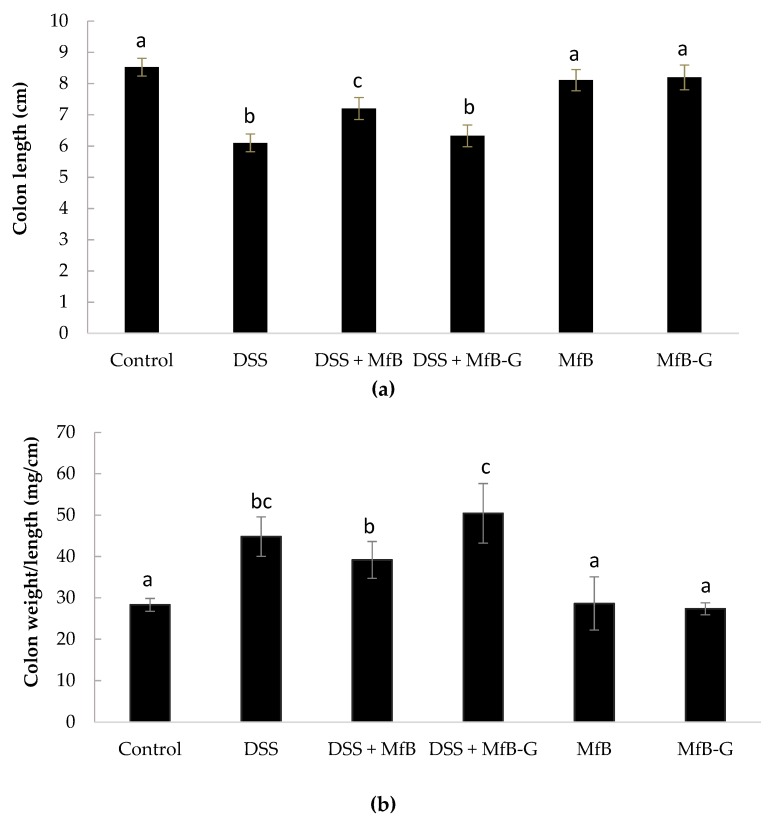
Evaluation of colon length (**a**) and presence of edema (**b**) after administration of plant sterol enriched milk-based fruit beverages without (MfB) or with galactooligosacharides (MfB-G) for 56 days on dextran sulphate sodium (DSS)- chronic colitis induction. Bars show the mean ± standard deviation (*n* = 8). Different lowercase letters (a–c) indicate statistically significant differences (*p* < 0.05) among samples.

Figure 3a shows that mice exposed to DSS (6.10 ± 0.28 cm), DSS + MfB (7.20 ± 0.35 cm) and DSS + MfB-G (6.33 + 0.35 cm), suffered a significant shortening (*p* < 0.05) of colon length in comparison to the control (8.53 ± 0.29 cm). DSS + MfB mice showed longer colon (18%) than DSS mice, significantly preventing (*p* < 0.05) the colon shortening induced by DSS. Mice that received DSS showed higher presence of edema in colonic tissue than the control group (39–50 vs. 28 mg/cm, respectively) (see Figure 3b). Administration of beverages did not show any significant (*p* > 0.05) protective effect with respect to edema development. Mice that received MfB and MfB-G showed very similar colon length and tissue edema compared to control, which is according with the absence of clinical symptoms observed on these mice groups.

### 3.3. Histopathological Analysis

**Figure 4:**
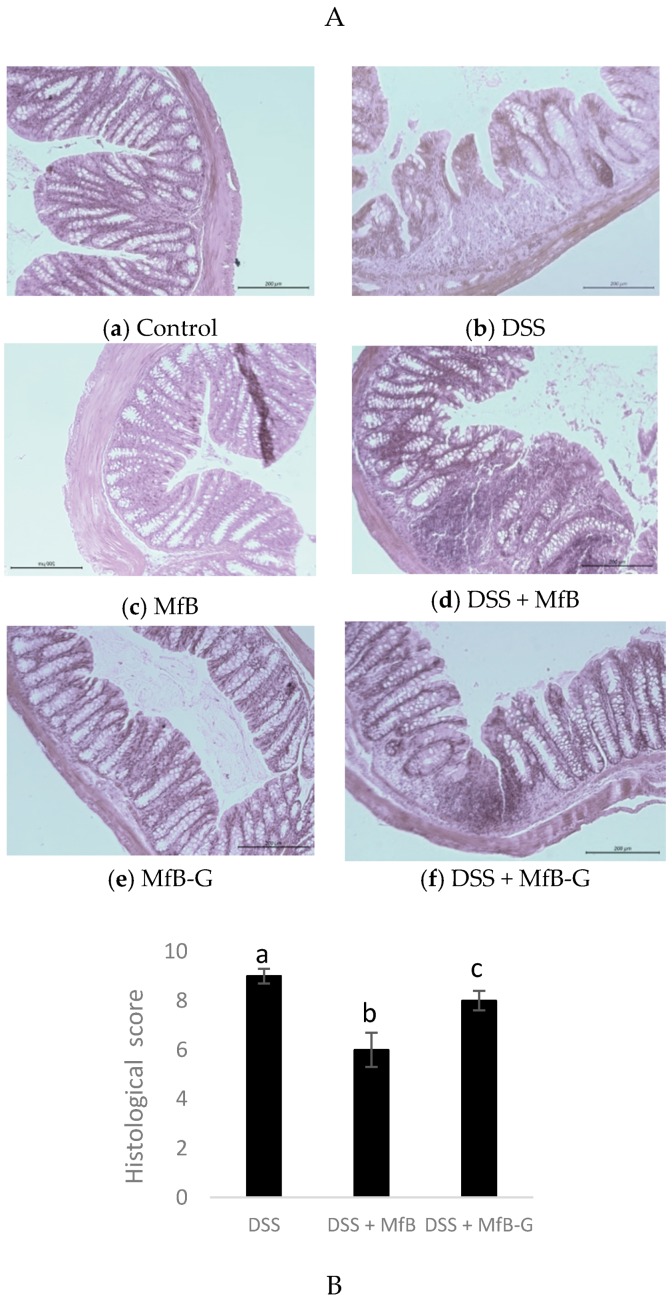
The representative photographs of hematoxylin and eosin staining (magnification ×200) (**A**) and the corresponding score (**B**) of colon tissues obtained after administration of plant sterol enriched milk-based fruit beverages without (MfB) or with galactooligosacharides (MfB-G) for 56 days on dextran sulphate sodium (DSS)- chronic colitis induction. Data are expressed as the means ± standard deviation of six mice in each group. Representative images of staining of colon tissues from each (*n* = 3) *p* < 0.05 vs. normal control. Different lowercase letters (a–c) indicate statistically significant differences (*p* < 0.05) among samples.

As is shown in Figure 4A, control, MfB and MfB-G mice groups (Figure 4a,c,e, respectively) had a morphologically normal colon without signs, or very low levels of leucocyte infiltration. In contrast, DSS group (Figure 4b) suffered a marked inflammation characterized by a loss of epithelial architecture, reduction of number of globet cells and crypts, as well as a strong increase of immune cell infiltration compared to control (Figure 4a), what it was reflected in a high histological score (9/10 points). Treatment with DSS + MfB (Figure 4d) or DSS + MfB-G (Figure 4f) mice showed similar histopathology alteration compared to DSS group (b), with immune cell infiltration and distortion of crypts, although the epithelial architecture was more preserved resulting in a significant (*p* < 0.05) lower histological score (34 and 10%, respectively) values. However, DSS + MfB-G (Figure 4f) mice showed a higher histological score (25%) and damage in the epithelial architecture, with distortion of crypts and high presence of immune cell in comparison to the DSS + MfB group (Figure 4d). 

### 3.4. Presence of MPO in Colonic Tissue

**Figure 5:**
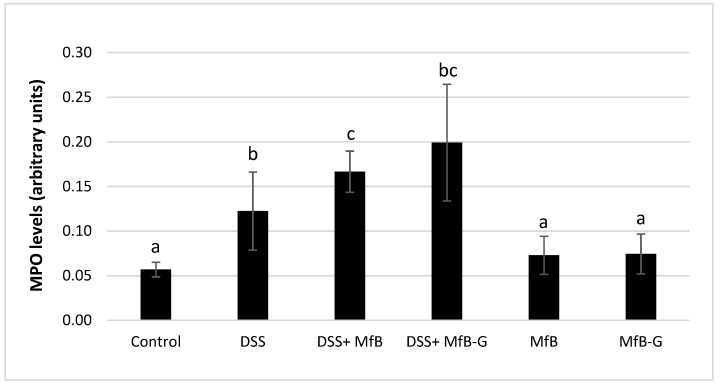
Presence of myeloperoxidase (MPO) levels in colonic tissue after administration of plant sterol enriched milk-based fruit beverages without (MfB) or with galactooligosacharides (MfB-G) for 56 days on dextran sulphate sodium (DSS)- chronic colitis induction. Bars show the mean ± standard deviation (*n* = 8). Different lowercase letters (a–c) indicate statistically significant differences (*p* < 0.05) among samples.

Similar to that observed in the histopathological analysis, mice receiving DSS showed high levels of immune cells infiltration, raising from 2 to 3.5-fold MPO levels with respect to control (see Figure 5). Daily administration of beverages (DSS + MfB or DSS + MfB-G groups) failed to attenuate the increase of MPO levels induced by DSS. MfB and MfB-G mice groups had very similar MPO values with respect to the control, suggesting that both beverages apparently do not trigger an inflammatory response in the colon in the absence of colitis.

## 4. Discussion

To investigate the potential anti-inflammatory effects of PS enriched milk-based fruit beverages with or without GOS on UC, a model of chronic colitis induced by DSS in mice was selected. Although colitis animal models do not represent the complexity of human disease, they provide valuable information about factors involved in the inflammatory process and allow to evaluate different therapeutic strategies to improve life quality of patients with IBD [38]. In particular, the DSS-induced colitis model can easily reproduce the acute and chronic phases or the relapsing periods typical of UC, depending on the concentration and cycles of exposition to DSS in drinking water. Moreover, this model exhibits similar symptoms to those of human UC (diarrhea, bloody feces and body weight loss) and histological features such as mononuclear leucocyte infiltration, crypt architectural disruption and epithelial degeneration [39]. 

Our study showed that daily administration of MfB beverage resulted in a significant (*p* < 0.05) reduction of symptoms associated to UC, mainly preventing the presence of diarrhea or alleviating their increase during all experiment (vs. DSS group), as well as, protecting against presence of bloody feces in the acute phase of the disease (first DSS cycle). Reduction of clinical symptoms led to partially prevention of colon shortening induced by DSS cycles, showing longer colons (18%) compared to DSS group. Histopathological analysis of the colon revealed a slight improvement in the architecture of the colonic epithelium and a greater number of crypts compared to the DSS group, in agreement with the previous parameters mentioned. However, the distribution and morphology of the crypts were altered, as well as an increase of neutrophil infiltration and a high MPO level in the colonic tissue was observed. Stimulation of neutrophil activity or its migration into colon tissue, could be related with the prevention of colon shortening observed after MfB treatment. Neutrophils are considered the first line of defence against microorganisms and recently, it has been demonstrated that they can build a complex formed by chromatin and neutrophil proteins that act as immunomodulator and activate immune cells such as T cells, although their specific role on IBD has not been well described yet [40]. Beneficial effect of MfB upon DSS-induced colitis can also be related with its cytoprotective effect at intestinal level. In a previous in vitro study by our research group, it was demonstrated that milk-based fruit beverages had several beneficial effects against oxidative stress and prevention of cell dead, inhibiting some important pro-apoptotic events and preserving cell monolayer integrity in a differentiated colon cancer (Caco-2) cell model [32]. This fact could be important because DSS is a toxic compound to colonic epithelial cells that cause an increase of apoptotic cells and compromise the epithelial barrier integrity through the loss of some important proteins present in the tight junctions [41]. 

It is important to note our study design does not allow knowing which bioactive compound/s contained in the beverages has the anti-inflammatory effect on DSS-induced colitis. Nevertheless, it may be possible that the specific combination of all of them produce the effect observed in our study. The presence of mandarin juice (represents almost half of MfB composition), could contribute to the anti-inflammatory effect since contains important quantities of antioxidant phytochemicals such as flavonoids (mainly hesperidin, narirutin and vicenin-2) and β-cryptoxanthin (β-Cx) [42]. In this sense, flavonoid-rich extracts (containing mainly hesperidin, narirutin and vicenin-2) obtained from blood orange juice administrated (40 mg/kg/day) to CD1 mice with colitis-induced by dinitrobenzene sulfonic acid, have shown preventive effects against the colonic pathological tissue damage. Flavonoids acted mainly counteracting NF-κB signalling, decreasing expression of pro-apoptotic proteins (Bax) and restoring the redox balance in colonic tissue [10]. Similar effects were observed in a recent study with industrial orange by-products (citrus pectin and different sub-fractions obtained from orange after juice extraction) in DSS-treated mice [11]. Byproducts with high polyphenol total content and antioxidant capacity showed better anti-inflammatory effect in terms of clinical symptoms and reduction of pro-inflammatory mediators’ expression (TNF-α, IL-1β, IL-6). On the other hand, the presence of β-Cx could contribute to the beneficial effects of the MfB, although its specific role on UC has not been studied yet. In steatohepatitis and insulin resistance murine models induced by high fat content diets, β-Cx administration (~2.5–7.5 mg/kg/day) lead to attenuation of lipotoxicity-induced inflammation, preventing hepatic tissue peroxidation (TBARS) and the macrophages activation [43], as well as the stimulation of antioxidant enzymes (catalase, superoxide dismutase and glutathione peroxidase) and inhibition of the expression of pro-inflammatory markers (NF-κB and TNFα) in liver [44]. Therefore, β-Cx is able to reduce the pro-inflammatory process through a direct and indirect anti-oxidant mechanism. Taking into account that oxidative stress has a pivotal role on UC, daily administration of MfB containing β-Cx (0.02 mg/kg/day) could help to mitigate the pro-inflammatory process. 

Additionally, it is important to note that enrichment of MfB with PS could be a remarkable factor in its anti-colitic effect, since several studies using PS standard solutions (β-sitosterol, stigmasterol and γ-oryzanol) added to feed and administered at doses between 20–50 mg/kg/day (similar to our study 35 mg/kg/day) for 3-56 days have shown a marked anti-inflammatory effect independently of the colitis animal model used. In C57BL/6 mice, β-sitosterol (20 mg/kg/day) administration for 56 days prevented the colon shortening (~8%) and reduced MPO activity in colon tissue (~35%), what led to a lower level of pro-inflammatory cytokines (IL-1β, TNF-α and IL-6) after colitis induction by high fat diet (60 Kcal% from fat) [16]. Similarly, β-sitosterol administration (10 or 20 mg/kg) during 3 days to C57BL/6 mice with colon inflammation induced by TNBS prevented partially colon shortening (~3.4%) and improved the pro-inflammatory status, reducing pro-inflammatory cytokine levels (IL-1β, TNF-α and IL-6) and MPO activity in colon tissue (~42%), with a concomitant inhibition of NF-κB translocation into the nucleus [17]. In UC models induced by DSS, administration to C57BL/6 mice of γ-oryzanol (50 mg/Kg/day for 16 days), a mixture of phytosteryl ferulates derived from rice bran oil, mitigated clinical symptoms associated to UC and partially prevented colon shortening (~9%) and the pathophysiological activity during colonic inflammation through inhibition of NF-κB activation after 5 days of DSS at 3% (v/v) induction [24]. Differences observed in terms of MPO activity with respect to our study, could be attributed to the fact that PS are added into the beverages as a food ingredient composed of a complex mixture of PS (β-sitosterol, sitostanol, campesterol, campestanol and stigmasterol). As far as we know, there are no studies that evaluate the anti-inflammatory effect—in murine chronic colitis models—of some of the PS found in the food ingredient used in the PS enrichment of our beverages (sitostanol, campesterol and campestanol), which suppose 20% of the total PS. The small molecular structural differences among sitostanol, campesterol or campestanol, in comparison to β-sitosterol, could explain the lack of beneficial effect observed on the pro-inflammatory process. Feng et al. [21] observed that β-sitosterol or stigmasterol administration (0.4% w/w) in C57BL/6J mice similarly mitigate inflammation severity and macroscopic damage (colon shortening and histopathology score) induced by DSS (1.5% *w*/*v*, for 5 days), but only stigmasterol was able to reduce the expression of cyclooxygenase-2. Authors indicate that the presence of a double-bond in the side chain in stigmasterol could be responsible for their additional anti-inflammatory effect compared to β-sitosterol. In this sense, different behaviour of β-sitosterol, campesterol and stigmasterol at the same concentration (24 µM for 48 h) and cell system (macrophages) have been observed. Stigmasterol suppressed cytokine secretion into the supernatant, while β-sitosterol promoted it and campesterol did not have any effect [45]. Moreover, it is also possible that some specific compounds present in our beverages hidden the beneficial effects of PS on colitis. Llewellyn et al. [46] observed that administration of a high casein diet (41% w/w) for 14 days promoted intestinal barrier damage and increased colonic cytokines levels (IL-6 and TNF-α) in DSS induced model in mice (3% *w*/*v* for 7 days). Although, the casein intake is around 850-fold higher than our study, the longer administration time of beverage (14 vs. 56 days) and DSS (7 vs. 56 days) exposition could explain our results. Besides, the potential impact of other compounds present in the beverage on the colitis process cannot be ruled out. 

Regarding the potential beneficial effect of GOS on DSS-induced colitis, results show that MfB-G administration during all the experiment does not confer additional beneficial effects with respect to MfB. DSS + MfB-G mice suffered a dramatic increase of clinical symptoms from the third DSS cycle, remaining constant up to the end of the study. This fact could explain the absence of a beneficial effect of MfB-G on colon shortening (12% shorter than MfB), the higher colonic mucosa alteration (distortion of crypts and high immune cells infiltration) and MPO level compared to MfB. The effect of GOS and mechanisms which could improve UC in murine colitis models have been poorly studied and currently are controversial. Holma et al. [30] reported that administration to rats of two kinds of GOS (whey and lactose derived from cow milk) at 4 g/kg/day for 10 days increased notably bifidobacterial number in feces, which are high producers of anti-inflammatory compounds, such as short-chain fatty acids. However, the increase of bifidobacterial number was not correlated with an improvement of colonic damage and they did not prevent immune cell infiltration (MPO activity) and edema induced by TNBS during 72 h. In contrast, in mice deficient in *smad-3* (phenotype characterized by colon moderate inflammatory response) infected by *Helicobater hepaticus*, GOS supplementation (5 g/kg/day) for 42 days reduced colitis severity preserving colon architecture by modulating the function and trafficking of natural killer cells [31]. The use of different animal species, agents of induction of colitis, treatment times and type of GOS could explain the differences found between the studies. However, it has been reported previously the harmful effect of fructooligosacharides (FOS) administration (at 6 g/day), a kind of soluble fibre similar to GOS, in rats with colitis induced by DSS (at 3% for seven days). Similar to our results, FOS slightly prevented symptoms associated to UC (13–45%, DAI value) at the beginning of the study but quickly worsened without showing differences with respect to DSS group up to the end of the study. FOS treatment did not prevent the colon shortening and exacerbated colon histological damage severity and MPO activity, as well as reduced crypt cell proliferation in the distal colon, which is an integral part of the colon repair process. Authors indicated that FOS delayed the onset of repair, promoting the harmful effect of DSS on colon epithelia [47]. Other possible hypothesis could be related with the bifidogenic effect of FOS, what lead to the increase of bifidobacterial genus in the colon. It has been indicated that a quick rate of FOS fermentation in the cecum produce an overproduction of organic acids as lactic and acetic acids [48], which can damage the intestinal epithelium, leading to an increase of colonic permeability [49]. Similar to FOS, it has been indicated that GOS fermentation (72 h) lead to a quick increase and accumulation of lactic and acetic acids in the colon, compared to propionic and butyric acids, in a dynamic in vitro colon model (TIM-2). These increases were correlated with an increase of bifidobacteria and lactobacilli genus [50]. Then, we hypothesized that MfB-G administration could produce a change in intestinal microbiota by increasing the organic acids that produce bacteria, which could exacerbate the loss of the epithelial barrier integrity and the colonic mucosal permeability induced by DSS. Moreover, the potential inhibitory effect of GOS on the colonic repair mechanism cannot be ruled out.

In summary, PS-enriched milk-based fruit beverage (MfB) shows a moderate anti-inflammatory effect, helping to alleviate the clinical symptoms associated to colitis, the colon shortening and colonic damage in a DSS-induced mice model of chronic colitis. Neutrophil infiltration in colonic tissue and MPO level remained higher after MfB treatment, suggesting that they could be involved in its anti-inflammatory action as a compensatory mechanism trying to overcome the damage induced by DSS. GOS addition to the MfB did not show any additional beneficial effects in comparison with MfB and even exacerbated the pro-inflammatory action induced by DSS. The higher DAI value, colonic mucosa damage, immune cell infiltration and MPO level, suggest that presence of GOS in colon compromise colonic epithelial permeability or delay the reparation colonic mechanism, promoting the cytotoxic effect of DSS on colon cells. 

Our results demonstrate the importance to evaluate the biological effects of bioactive compounds in the context of a complex food matrix. PS-enriched foods could be a suitable strategy to extend remission periods and improve the quality of life of patients with UC, but further investigation is needed to confirm the beneficial role of PS in a food matrix on the UC. 

## Figures and Tables

**Table 1:** Scoring system to calculate the disease activity index (DAI *).

Score	Weight Loss (%)	Stool Consistency	Visible Blood in Feces
0	None	Normal	None
1	1–5		
2	6–10	Loose	Slight bleeding
3	11–20		
4	<20	Diarrhea	Gross bleeding

* DAI was calculated by combining scores (WS: weight loss; SC: stool consistency; BF: bloody feces).

**Table 2:** Scoring system used to calculate the histological score in dextran sulphate sodium (DSS)-induced colitis.

Histological Scoring System for DSS-Induced Colitis		
Feature	Score	Description
Severity of inflammation	0	None
	1	Mild
	2	Moderate
	3	Severe
Extent of inflammation	0	None
	1	Mucosa
	2	Mucosa and submucosa
	3	Transmural
Crypt damage	0	None
	1	1/3 damages
	2	2/3 damaged
	3	Crypts lost, surface and epithelium present
	4	Crypt and surface epithelium lost

Histological score is calculated by adding the score of three parameters up to a total score of 10 points. DSS: dextran sulphate sodium;
